# Epstein–Barr Virus and Human Papillomaviruses Interactions and Their Roles in the Initiation of Epithelial–Mesenchymal Transition and Cancer Progression

**DOI:** 10.3389/fonc.2018.00111

**Published:** 2018-05-01

**Authors:** Farhan S. Cyprian, Halema F. Al-Farsi, Semir Vranic, Saghir Akhtar, Ala-Eddin Al Moustafa

**Affiliations:** ^1^College of Medicine, Qatar University, Doha, Qatar; ^2^Biomedical Research Centre, Qatar University, Doha, Qatar; ^3^Oncology Department, McGill University, Montreal, Quebec, Canada

**Keywords:** Epstein–Barr virus, high-risk human papillomaviruses, onco-proteins, epithelial–mesenchymal transition, cancer progression

## Abstract

Oncoviruses are implicated in around 20% of all human cancers including both solid and non-solid malignancies. Epstein–Barr virus (EBV) and human papillomaviruses (HPVs) are the most common oncoviruses worldwide. Currently, it is well established that onco-proteins of EBV (LMP1, LMP2A, and EBNA1) and high-risk HPVs (E5 and E6/E7) play an important role in the initiation and/or progression of several human carcinomas, including cervical, oral, and breast. More significantly, it has been recently pointed out that viral onco-proteins of EBV and high-risk HPVs can be co-present and consequently cooperate to initiate and/or amplify epithelial–mesenchymal transition (EMT), which is the hallmark of cancer progression and metastasis. This could occur by β-catenin, JAK/STAT/SRC, PI3k/Akt/mTOR, and/or RAS/MEK/ERK signaling pathways, which onco-proteins of EBV and HPVs share. This review presents the most recent advances related to EBV and high-risk HPVs onco-proteins interactions and their roles in the progression of human carcinomas especially oral and breast *via* the initiation of EMT.

## Introduction

Today, it is well-established that lifestyle, gene alteration in addition to infections from microorganisms are important risk factors for human oncogenesis. Accordingly, it was revealed that more than 50% of malignancy cases are associated with preventable origins, including oncoviruses infections (1). Globally, cancer cases associated with infections is around 20%; as roughly, two million of new malignancies reported in humans are linked with pathogens; among them 1.6 million occur in developing countries. More than two-thirds of malignancy cases are associated with well-characterized oncogenic viruses, including Epstein–Barr virus (EBV) and human papillomaviruses (HPVs) (2, 3).

Carcinogenic properties of oncoviruses are determined based on their capability to provoke cellular transformation and consequently tumor development; an effect that is attributed to genetic deregulation of infected cells leading to alteration of their normal functions. For instance, it is well-established that EBV and high-risk HPVs onco-proteins can take over intracellular and extracellular signaling pathways, provoke genomic instability, increase the life-span of infected cells (by inhibiting apoptosis), and destabilize cell senescence process, resulting in uncontrolled cell proliferation (3). These elements are important biological features of carcinogenesis (4), which can be provoked following infection by oncoviruses, including EBV and high-risk HPVs.

On the other hand, it has been established that the epithelial–mesenchymal transition (EMT) event is an important physiological procedure in the development of metastatic cancer (5). Likewise, it has been pointed out that onco-proteins of EBV (LMP1, LMP2A, and EBNA1) and high-risk HPVs (E5 and E6/E7), can enhance cancer progression of human carcinomas *via* the initiation of EMT (6, 7). Meanwhile, it is important to highlight that EBV and high-risk HPVs can be co-present in certain types of human malignancies especially oral and breast cancer (8, 9); consequently, onco-proteins of these viruses can cooperate to increase invasive ability of such cancers *via* the “amplification” of EMT. This review consolidates the existing evidence on putative effects of the co-presence of EBV and high-risk HPVs and their association with EMT and human carcinomas, especially oral and breast, in order to explain the conceptual framework for the impact of co-viral infection in cancer progression.

## EBV and EMT in Human Carcinomas

Epstein–Barr virus is a very common human gammaherpesvirus, as roughly more than 90% of the adult population is infected by this virus at one point of their life (10). Acute infection with EBV can cause infectious mononucleosis (glandular fever), and its latent state can evolve to yield several B-cell lymphomas, oral cancers (especially nasopharyngeal carcinomas: NPC), gastric cancer, and other malignancies (11, 12). EBV-infected cells express six EBV nuclear antigens (EBNA1, -2, -3A, -3B, -3C, and -LP) in addition to three latent membrane proteins (LMP1, -2A, and -2B), and multiple non-coding RNAs (EBERs and miRNAs) (13–15).

The expression patterns of these genes define the types of cancers correlated with EBV (11, 12). For example, type II latency which is associated with LMP1, -2A, and EBNA1 gene expressions is linked with Hodgkin’s lymphoma and nasopharyngeal as well as other carcinomas, including gastric and probably breast (16–18). Thus, LMP1 is regarded as the main EBV-encoded oncogenic protein as it induces a multitude of effects promoting cell growth, protecting cells from apoptosis, enhancing cell motility, and stimulating angiogenesis; additionally, it is frequently expressed in EBV-associated human oral carcinomas (18, 19). Several recent studies including two from our lab revealed that EBV is present in around 40% of human breast cancer samples and its presence is associated with more aggressive phenotypes (9, 20–26).

Regarding the interaction between EBV onco-proteins and EMT, it has been revealed that LMP1 can trigger multiple signaling pathways, including NF-κB, PI3K/Akt, and MAPK, all of which are actively involved in the induction of EMT (7, 27, 28). Accumulating evidence has shown that LMP1 can downregulate E-cadherin expression (27, 29) by inducing a transcriptional repression complex composed of DNA methyltransferase and histone deacetylase, which is located on the E-cadherin gene promoter (*CDH1* gene). LMP1 can also stimulate the exchange from E-cadherin to N-cadherin; and enhance the association of β-catenin with N-cadherin (30). Furthermore, LMP1 stimulates the expression of metalloproteinase 9 and regulates the transcription factors TWIST, SNAIL, and β-catenin (28, 31, 32).

On the other hand, LMP2A is another onco-protein of EBV and is overexpressed in the vast majority of EBV-associated carcinomas, especially NPC (33). It has been shown that LMP2A augments the invasive/migratory ability and incites changes in EMT-like cellular biomarkers (34); additionally, the same authors pointed out that LMP2A can induce EMT initiation by activating the 4EBP1–eIF4E axis thereby enhancing the expression of metastatic tumor antigen-1 by targeting the rapamycin (mTOR) pathway.

EBNA-1 onco-protein of EBV has a multifunctional role as a virus-related protein. EBNA-1 is overexpressed in NPC, inducing higher invasion and metastatic ability, as well as influencing EMT biomarkers (35, 36); EBNA1 regulates EMT through the de-regulation of SLUG, SNAIL, TCF8/ZEB1, vimentin, occludins-1, as well as E-cadherin, which are important genes associated with EMT (36).

Finally, it is important to underline that miRNAs, as posttranscriptional regulators, are integrated into the EBV-regulated EMT program and consequently cancer progression (7, 37). So far, a total of 25 EBV miRNA precursors with 44 mature miRNAs have been classified and mapped to the BHRF1 and BART regions (4 and 40 miRNAs, respectively) of the EBV DNA (38). miR-BART9 is overexpressed in NPC and has been found to stimulate its metastatic ability by targeting E-cadherin and inducing a mesenchymal phenotype and biomarkers (39). Recently, it has been reported that targeting PTEN 3′UTR, miR-BART7-3p downregulates epithelial biomarkers, and persuades mesenchymal features *via* PI3K/Akt/GSK-3 signaling pathways; this can lead to a high expression and nuclear accumulation of Snail and β-catenin in NPC and associates positively with lymph node metastasis (40).

Aga et al. (41) reported that treatment of EBV-negative cells with LMP1-exosomes increases migration and invasiveness of NP cell lines, which correlates with phenotypes associated with EMT. He et al. (42) pointed out that miR-BART6-3p, which is an EBV-encoded microRNA, inhibits EBV-associated cancer cell migration and invasion of NPC and gastric cancer cells by reversing the EMT event. On the other hand, a recent investigation by Zuo et al. (43) revealed that cadherin 6 is upregulated in LMP1-positive NPC tissues, which is identified as a target of the epithelium-specific miR-203. While, cadherin 6 activation in turn can induce EMT and promote metastasis in NPC. Moreover, it has been recently indicated that the most abundant miRNAs of EBV, in gastric cancer, are Bart4, Bart11, Bart2, Bart6, Bart9, and Bart18. Among them, Bart9 displays the same sequence as hsa miR-200a and miR-141; while, BART9 knockdown can enhance E-cadherin expression in EBV-positive gastric cancer cells (44). Taken together it implicates EBV infection in EMT initiation and consequently cancer progression, especially oral, *via* its onco-proteins and non-coding RNAs; however, we believe that more investigations are necessary to understand the role of all EBV onco-proteins and miRNA in the initiation of EMT in human carcinomas, especially breast.

## High-Risk HPVs and EMT in Human Malignancies

HPVs are small, double-stranded DNA viruses that mostly infect cutaneous and mucosal epithelial tissues of the anogenital tract. Over 150 different types of HPV have been identified so far, one-third of which infect epithelial cells in the genital area (6). HPVs are classified as high or low risk, where high-risk types are linked with cancer development, while low-risk types are generally self-limiting and do not cause cancer. In contrast, infections with high-risk HPVs (type 16, 18, 31, 33, 35, 39, 45, 51, 52, 55, 56, 58, 59, 68, 73, 82, and 83) are correlated mainly with cervical cancers, as approximately 96% of these malignancies are positive for high-risk HPVs (45–49). More specifically, high-risk HPV early proteins, or onco-proteins, including the E5, E6, and E7 increase cellular alterations that can possibly lead to HPV-induced carcinogenesis (6, 50, 51). In this regard, earlier studies demonstrated that the E5 onco-protein could affect cellular transformation and consequently lead to carcinogenesis *via* its interaction with EGF-R1 signaling pathways (MAP kinase and PI3K/Akt) and pro-apoptotic proteins (52–54).

E6 and E7 of high-risk HPVs are assumed to work together, as they are both expressed from bicistronic mRNA (55) and initiated from the viral early promoter (p97). E6 and E7 both have functions that affect cell cycle progression due to their association with cell cycle controllers (50, 56, 57).

The viral E7 onco-protein causes an unscheduled S-phase entry which is complemented by the role of E6 that prevents the induction of apoptosis (58). Alternatively, it has been shown that the interaction of E6 with p53 leads to the inactivation of p53-mediated growth suppression and/or apoptosis (59). Also, E6 can associate with other pro-apoptotic proteins, including Bak and Bax (60–62). Nevertheless, the E6 onco-protein of high-risk HPVs can enhance cell proliferation independently of E7 through its C-terminal PDZ-ligand domain (63), which mediates suprabasal cell proliferation (64, 65) and may lead to cancer progression by disrupting normal cell–cell adhesion. On the other hand, several investigations have documented the correlation between E7 with members of the pocket protein family, such as pRb. This connection prevents S-phase entry by displacing E2F family of transcription factors (56), irrespective of the presence of external growth factors, causing the expression of DNA replication proteins (55, 66).

Concerning the role of high-risk HPVs in cancer progression and EMT, it is well-known that onco-proteins of these viruses are consistently expressed in infected carcinoma cells (67); this could form an important element to initiate cellular transformation and, therefore, tumor formation of certain types of malignancies *via* their involvement in the EMT process (68, 69). For instance, E6/E7 onco-proteins of HPV type 16 activate *Jagged1*, which can be associated with the induction of PI3K-mediated EMT. In addition, E6/E7 apparently incite FGF-induced EMT in cervical oncogenesis (70). In parallel, it has been shown that E6/E7 onco-proteins suppress the expression of E-cadherin in cervical cancer cells triggered by FGF stimulation, and consequently increase the invasiveness of cancer cells (70). On the other hand, it was reported that E6/E7 can induce EMT *via* PI3K/AKT and/or MEK/ERK in primary human keratinocytes (71); also, E6/E7 promote EMT *via* the activation of its transcriptional factors especially ZEB1 and ZEB2 (72).

In our laboratory, we have generated a novel model to explore the interaction outcome between E6/E7 onco-proteins of high-risk HPV and HER-2/ErbB-2 receptor in human head and neck (HN) carcinogenesis; this model was developed since ~25–30% of human HN cancers are positive for HPVs and express/overexpress HER-2 (73). Using this model, we reported that E6/E7 onco-proteins of HPV type 16 cooperate with HER-2 receptors to provoke cell transformation of human normal oral epithelial cells; this is accompanied by a delocalization of β-catenin from the undercoat membrane to the nucleus in these cells. The E6/E7/HER-2 cooperation also induces morphologic changes from a cobblestone-shaped epithelial to the spindle-shaped mesenchyme form, which enhances cell invasion and metastatic ability of these transformed cells. Additionally, our studies revealed that cyclin D1 is the main target of E6/E7/HER-2 interaction *via* the alteration of β-catenin’s role from a cell–cell adhesion protein to a transcriptional controller (73). Also, we have shown that cyclins D1, D2, and D3 are crucial for cell transformation provoked by E6/E7/HER-2 cooperation in our cell models and mouse normal embryonic fibroblast cells (74, 75). Last, our data pointed out that the cooperation outcome of E6/E7 and HER-2 takes place *via* β-catenin activation through pp60 (c-Src) phosphorylation (76).

Likewise, in oral cancer samples, it has been shown that E-cadherin is downregulated in HPV-positive samples in comparison with HPV-negative ones, while, vimentin expression remained unaltered (69); herein, it is important to highlight that both E-cadherin and vimentin are important biomarkers of EMT (5). In addition, Wakisaka et al. (77) reported that HPV-positivity is associated with EMT phenotype of oropharyngeal carcinomas and lymph node metastasis. Additionally, in tonsillar carcinoma cases, HPV-positivity is correlated with downregulation of E-cadherin and nuclear translocation of β-catenin indicating a more aggressive phenotype and risk of metastasis (78).

Next, to identify the role of high-risk HPVs infection in human cancer progression, we assessed the outcome of E6/E7 onco-proteins of HPV 16 in two non-invasive human breast cancer cell lines, MCF7 and BT20. Our data showed that E6/E7 of HPV 16 provoke cell invasive and metastatic capabilities of both cell lines (79). This is associated with an upregulation of Id-1, a family member of helix-loop-helix transcription factors, which is an important regulator of invasion and metastasis of breast cancer (80, 81). We further established that E6/E7 onco-proteins can enhance Id-1 promoter activity in both cancer cell lines. Our study on tissue samples indicated that HPV type 16 presence is significantly higher in invasive breast carcinomas in comparison with ductal carcinoma in *in situ* and normal breast tissues. Furthermore, our results displayed that Id-1 upregulation is associated with the presence of high-risk HPVs in human invasive and metastatic breast cancer tissues from Canadian and Syrian women (79, 82, 83). Herein, we would like to mention that the presence of high-risk HPVs in human breast cancers varies from 2 to 83% (please refer to the next section).

Concerning the role of E5 onco-protein of high-risk HPV and cancer progression, it is important to highlight that there are few investigations related to this critical topic (84, 85). However, it is evident that E5 can enhance cancer progression alone *via* its interaction with EGF-R1 signaling pathways (MAP kinase and PI3K/Akt) or *via* switching FGFR2b to FGFR2c (52–54, 85). In addition, it has been recently proposed that E5 of high-risk HPVs can cooperate with E6/E7 onco-proteins to enhance cancer progression *via* EMT (6).

Finally, it is important to highlight that recent studies have identified and validated HPV-encoded miRNAs (86). Accordingly, Liu et al. (87) reported that miR-375 deregulation can affect cell invasion ability of E6/E7-expressing cervical cancer cells *via* the modulation of EMT. Moreover, it has been revealed that E6 of HPV 16 can promote EMT and invasion in cervical cancer *via* the repression of miR-218 (88). On the other hand, it has been pointed out that E6 deregulate miR-34a, in HN cancer cells, which is an important controller of EMT and, therefore, cancer progression (89). Thus, it is evident that HPV-miRNA can play an important role in the regulation of cell invasion and metastasis *via* EMT.

Altogether these findings support the idea that high-risk HPVs can enhance cancer progression *via* the initiation of EMT.

## EBV/HPVs Interaction and EMT in Human Cancer

It is evident that some organs and tissues can be co-infected with more than one species of viruses, including EBV and HPVs. Accordingly, in 2009, we hypothesized that human oral normal epithelial cells, particularly nasopharyngeal tissues, are prone to persistent HPVs and EBV co-infections; hence, high-risk HPVs and EBV co-infections could have a major role in the initiation and/or progression of oral cancer (8). Several investigations have explored this avenue and showed a co-presence of EBV and HPV in different types of carcinomas, including cervical, oral (nasopharyngeal), and breast cancer, as well as other malignancies (90–93). Herein, we must underline that EBV or high-risk HPVs infection alone is not enough to initiate cellular transformation of normal epithelial cells; the infected cells must endure additional genetic changes, and/or co-infection with more than one type of oncovirus to reach a full transformation (9, 73–75). Thus, we have generated a new model to study the cooperation effect between high-risk HPVs and HER-2 receptor in HN oncogenesis; as approximately 25 and 30% of HN cancers overexpress HER-2 and are positive for high-risk HPVs (73). As we mentioned above, we found that E6/E7 onco-proteins of HPV 16 cooperate with HER-2 to provoke cellular transformation and initiate EMT of human normal oral epithelial cells (73, 74).

**Figure 1:**
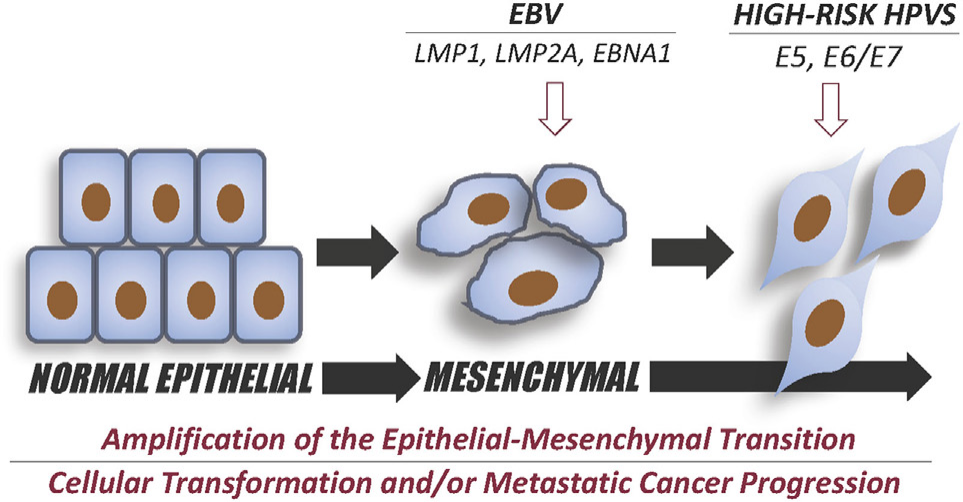
Diagram summary of the effect of Epstein–Barr virus EBV and high-risk human papillomaviruses (HPV) co-infection in the initiation and intensification of epithelial–mesenchymal transition (EMT) event. EBV onco-proteins, LMP1, LMP2A, and/or EBNA1 can initiate EMT and, therefore, cancer progression; while, co-expressing E5 and/or E6/E7 of high-risk HPV onco-proteins could enhance the progression of EMT leading to a more aggressive metastatic cancer, as previously reported by Al Moustafa et al. (9) and Jiang et al. (95).

In order to further explore the prevalence of EBV and high-risk HPVs in human HN cancers including oral malignancy in the Syrian population, we examined the presence of these viruses in a cohort of 80 oral cancer tissue samples from Syria using immunohistochemistry and Tissue Microarray methodologies. Our data revealed that 43% of these cancers are positives for high-risk HPVs (48, 49, 83). Genotyping investigation of high-risk HPVs showed that HPV types 16, 18, 31, 33, and 35 are the most frequent HPV types in HN cancers in Syria (94). The co-presence of EBV and high-risk HPVs in these samples is currently under investigation. In parallel, and in collaboration with our colleagues (Drs. Alaoui-Jamali and da Silva from McGill University), we are exploring the co-prevalence of EBV and high-risk HPVs in Canadian oral cancer samples. While, presently, there are no studies vis-à-vis the mechanisms of EBV and HPVs onco-proteins interactions in human oncogenesis; however, we believe that EMT initiation and amplification is the main target of EBV/HPVs interaction in human carcinogenesis (Figure 1). Thus, in our laboratory, we are presently exploring this important topic using both human normal oral and mammary epithelial cells as well as cancer cells.

Meanwhile, few studies have correlated the presence of EBV with HPV in human oral squamous cell carcinomas (SCCs). For instance, in oropharyngeal cancer, the presence of EBV and HPV viruses together in approximately 15–20% of oral SCCs (91, 96).

Likewise, Jiang et al. (89), found that 75% of tonsillar carcinomas and 90% of tongue SCCs are HPV-positive. However, EBV alone was found in 42 and 80% of tonsillar and tongue SCCs, respectively. In parallel, EBV and HPV co-infection was observed in 25% of tonsillar and 70% of tongue SCCs (95). Herein, it is important to emphasize that the presence of EBV or HPVs in NPC and/or oral SCCs is correlated with an overall better survival compared to EBV or HPVs-negative cancers (97–99). This may be attributed to the possible role of onco-proteins of these viruses, especially in the case of HPVs, in making cancer cells more sensitive to chemotherapy (100). However, it has been demonstrated that EBV and HPV co-infections can enhance invasiveness ability of human oral cancer (89) and breast cancer, as described in the next paragraph.

Concerning EBV and HPVs in human breast cancer, it has been shown that around 30–50% of breast malignancy cases are positive for EBV (21–25). In contrast, few studies were unable to detect EBV in human breast carcinomas (101, 102). In our lab, we explored the prevalence and role of EBV infections in human breast carcinogenesis; our investigation showed that around 52% of our samples are positive for EBV. We also noted that the presence of EBV is associated with invasive breast cancer phenotype in more than 60% of the examined cases (26).

Earlier studies showed that HPVs could be found in 2–86% of human breast cancer cases (24, 82, 92, 103–105). However, a small number of investigations could not find HPVs in breast cancer and normal mammary tissues (106–108). In this regard, E6/E7 onco-proteins of HPVs have been identified in breast cancer (109); though, there is a low level of transcription of these onco-proteins (110). Meanwhile, it has been reported that the presence of HPVs in breast cancer is associated with more aggressive phenotype (9, 82, 103, 111).

Previous studies predicted that oncoviruses, including EBV and high-risk HPVs, can be co-present in human breast cancer and consequently can play critical roles in the initiation and/or progression of this cancer (24, 92, 112, 113). To explore the co-presence and cooperation effect of EBV and high-risk HPVs in human breast cancer, we investigated their co-presence in breast cancer samples from Syria. We found that 32% of our samples are positive for both high-risk HPVs and EBV. Additionally, we examined the association between the co-existence of these viruses and cancer phenotype. Our data pointed out that the co-presence of EBV and HPVs is linked with high-grade invasive ductal carcinomas and lymph node involvement (9).

On the other hand, we would like to mention that, in this current issue, Vranic et al. (114) as well as de Lima et al. (90) reviewed the prevalence and role of EBV/HPVs co-infection in human cervical cancer. They pointed out that ~29% of human cervical carcinomas are positive for both EBV and HPVs, which is associated with an invasive cancer phenotype.

**Figure 2:**
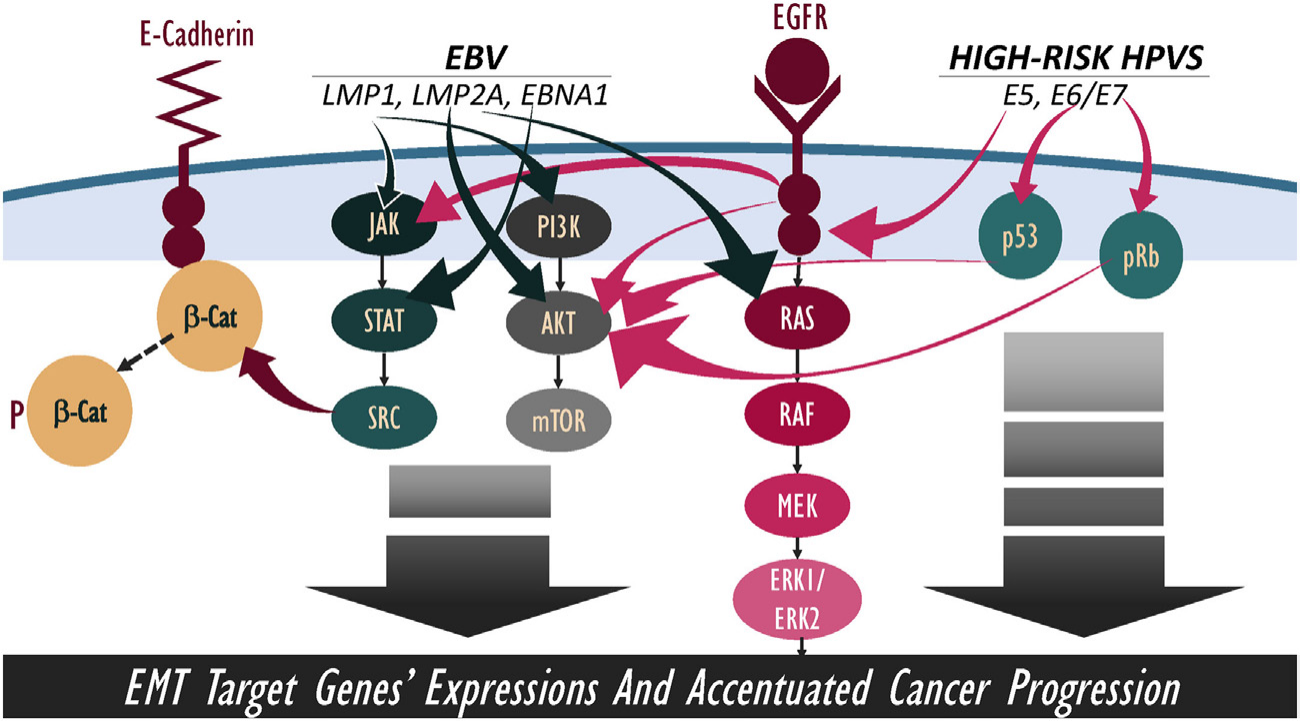
Schematic outline showing potential cooperation between Epstein–Barr virus (EBV) and high-risk human papillomaviruses (HPVs) onco-proteins in the amplification of epithelial–mesenchymal transition (EMT) event. We note that EBV and high-risk HPVs onco-proteins share various downstream-signaling pathways, including β-catenin, JAK/STAT/SRC, PI3k/Akt/mTOR, and RAS/MEK/ERK; thus, pathways’ crosstalk of EBV/HPVs onco-proteins can lead to a more hostile metastatic cancer.

Overall, several studies as well as ours clearly indicate that oncoviruses, including EBV and high-risk HPVs, can be found in several human carcinomas, such as oral, breast, and cervical. We believe that their co-infection can have critical roles in the development of these malignancies and their progression; especially, since EBV and HPVs onco-proteins share several signaling pathways, such as β-catenin, JAK/STAT/SRC, PI3k/Akt/mTOR, and/or RAS/MEK/ERK, which can enhance cancer metastatic progression *via* the amplification of EMT (Figure 2). Thus, we think that the activation of these four pathways together could be the main mechanism behind the amplification of EMT (Table 1). Meanwhile, it is important to emphasize that co-infection of EBV and HPVs as well as other human viruses, such as herpes simplex virus 1 and 2, human cytomegalovirus, BK virus, JC virus, and adeno-associated virus, could also play a significant role in the development and/or progression of certain types of human carcinomas; this could involve other “unknown” mechanisms related to these co-infections (99). Nevertheless, it is important to highlight that there are no mechanistic studies regarding the role of EBV/HPV viral co-infection and the EMT event.

**Table 1:** Epstein–Barr virus (EBV) and high-risk human papillomaviruses (HPVs) onco-proteins interactions can occur *via* β-catenin, JAK/STAT/SRC, PI3k/Akt/mTOR, and/or RAS/MEK/ERK signaling pathways and probably other paths.

The most common pathways of EBV and high-risk HPVs	
*HR-HPV Onco-proteins*	E5→EGFR→RAS→RAF→MEK→ERK1/ERK2
	→AKT→mTOR
	→JAK→STAT→SRC
	→β-catenin
	E6→p53→AKT→mTOR
	E7→pRb→AKT→mTOR

*EBV Onco-proteins*	LMP1→JAK→STAT→SRC
	→β-catenin
	→PI3K→AKT→mTOR
	LMP2A→RAS→RAF→MEK→ERK1/ERK2
	→AKT→mTOR
	EBNA1→STAT→SRC
	→β-catenin

## Conclusion and Future Objectives

This review presented evidence that oncoviruses co-infection, including EBV and high-risk HPVs, are important factors in human oncogenesis, thus, it is clear that they can enhance the progression of human carcinomas *via* the initiation of EMT which could occur by β-catenin, JAK/STAT/SRC, PI3k/Akt/mTOR, and/or RAS/MEK/ERK pathways. Therefore, further studies are necessary to identify the exact signaling pathways of EBV/HPVs onco-proteins’ interactions with the EMT event, given that no studies are currently available on this topic. Meanwhile, we assume that generating new *in vitro* and, *in vivo* models, as in cell lines and animal ones are important to determine the exact roles of these oncoviruses together and to discern their functions in the initiation and/or progression of oncogenesis; this could provide new targets to manage the malignancies associated with these oncoviruses and their co-incidence.

Alternatively, and regarding the prevention of oncoviruses-associated cancers, we believe that the elimination of some known risk factors related to lifestyle can reduce the development of these malignancies and metastases; especially, since it has been pointed out that oncoviruses co-infection could play an important role in the progression of these cancers. Meanwhile, prevention of EBV and HPV co-infections using the upcoming and/or presently available vaccines, respectively (115–117), could significantly decrease the rate of EBV and HPVs-associated malignancies and their progression to invasive forms that are responsible for most cancer-related deaths.

## Author Contributions

FC, HA-F, SV, and SA edited the paper. A-EM wrote the paper from conception to its finalized form.

## Conflict of Interest Statement

The authors declare that the research was conducted in the absence of any commercial or financial relationships that could be construed as a potential conflict of interest.
